# Endoscopic endonasal surgery for non-invasive pituitary neuroendocrinology tumors with incomplete pseudocapsule

**DOI:** 10.3389/fneur.2023.1109388

**Published:** 2023-03-27

**Authors:** Gang Zhang, Pan Wang, Junwei Wang, Dewei Zou, Hui Yao, Jie Liu, Chao Tang, Haotian Jiang, Xiaorong Tan, Nan Wu

**Affiliations:** ^1^Department of Neurosurgery, Chongqing General Hospital, Chongqing, China; ^2^Chongqing Medical University, Chongqing, China; ^3^Department of Pathology, Chongqing General Hospital, Chongqing, China

**Keywords:** endoscopic endonasal surgery, intensive excision, pseudocapsule, pituitary neuroendocrinology tumors, pseudocapsule-based extracapsular resection

## Abstract

**Background:**

Pituitary neuroendocrinology tumors (PitNETs) with pseudocapsule can be effectively removed by the pseudocapsule-based extracapsular resection technique. In the areas without pseudocapsule, the tumor cells can spread into the adjacent tissues at the cellular level, which brings a great challenge to achieving total tumor resection.

**Methods:**

Our surgical strategy for PitNETs with an incomplete pseudocapsule is to combine the pseudocapsule-based extracapsular resection technique with the intensive excision technique for the removal of the tumor. Specifically, the pseudocapsule-based extracapsular resection technique is applied in the areas with pseudocapsule, while in the areas without pseudocapsule, the intensive excision technique bounded by adjacent normal structures is adopted. Moreover, a pathological examination was performed to determine the situations of pseudocapsule and tumor cell remnant.

**Results:**

All growth hormone-secreting PitNETs achieved biochemical remission after surgery. There was no deterioration of pituitary functions postoperatively, and the preoperative hypopituitarism had improved in all patients postoperatively. In total, two cases suffered a transient diabetes insipidus, and intraoperative cerebrospinal fluid leakage was observed in two cases but no postoperative cerebrospinal fluid leakage in all cases. There was no recurrence during the follow-up. The fragmental pseudocapsule and small tumor remnants were found in the majority of suspicious tissues by histological staining.

**Conclusion:**

The effectiveness and safety of the surgical strategy were preliminarily explored for removing PitNETs without incomplete pseudocapsules. In overview, the pseudocapsule-based extracapsular resection technique is applied in areas with pseudocapsule, while the intensive excision bounded by adjacent normal structures is adopted in other areas.

## Introduction

Endoscopy can provide a clear panoramic field of view and high-brightness lighting and observe hidden corners through angle lenses. Therefore, there is an increasing trend toward endoscopic endonasal surgery (EES) for pituitary neuroendocrinology tumor (PitNET) treatment (1–3). Current research suggests that the membranous layers surrounding a PitNET generally include the dural envelope, pituitary capsule, and pseudocapsule (4–6). The pseudocapsule is developed by a PitNET gradually compressing the adjacent normal pituitary gland and is not a true tumor capsule (6). According to the presence and degree of development of a pseudocapsule, PitNETs can be divided into three types: complete pseudocapsule, incomplete pseudocapsule, and no pseudocapsule. Moreover, Chen et al. discovered PitNETs with incomplete pseudocapsule are more common than complete pseudocapsule ones (7). For PitNETs without pseudocapsule, tumors were usually removed in a piecemeal fashion similar to internal decompression (8). For PitNETs with complete pseudocapsule, the pseudocapsule-based extracapsular resection technique has been recommended to achieve total tumor resection and maximize the prognosis effectiveness (9, 10). Unfortunately, this surgical technique is not available for the gross total resection of PitNETs with incomplete pseudocapsule. The previous literature reported that the tumor cells of PitNETs with incomplete pseudocapsule can spread into the adjacent tissues at the cellular level (11). There is no consensus on whether and how to remove the suspicious tissues of no pseudocapsule regions. In 2019, Nagata et al. suggested that the pituitary gland should be peeled-off after selective adenomectomy to remove a small tumor cell remnant in the adjacent pituitary gland (12), while some authors believe that resection without compromising pituitary function is imperative for patients, which is beneficial to the ultimate health outcome (13). In this study, we report 10 cases of PitNETs with incomplete pseudocapsule treated by EES and aim to analyze the efficacy and significance of the surgical strategy, that is, to use the pseudocapsule-based extracapsular resection technique in the areas with pseudocapsule, while in other areas, the intensive excision bounded by adjacent normal structures is adopted.

## Methods

### Patients

In a retrospective review of 10 patients with PitNETs, seven cases of non-functioning PitNETs and three cases of growth hormone (GH)-secreting PitNETs underwent EES from August 2019 to May 2020. According to the relationship between tumor and cavernous sinus (knosp grade), PitNETs are divided into grade 0, grade 1, grade 2, grade 3, and grade 4 (14). Generally, grade 3 and grade 4 PitNETs were considered to be invasive. Meanwhile, all patients underwent a standard preoperative hormone examination to confirm the preoperative clinical diagnosis and evaluate the preoperative anterior pituitary function based on published guidelines (15). In particular, the postoperative evaluation of GH-secreting PitNETs is very important, whose postoperative biochemical remission is defined as a random postoperative serum GH level of <1 μg/L (16). All patients underwent hormone examination at least 3 days, 1–2 weeks, 3 months, and one time per year thereafter to comprehensively evaluate the early and long-term pituitary function. Gross total tumor resection is defined as no residual tumor under intraoperative neuroendoscopy and a complete absence of abnormal enhancement on postoperative magnetic resonance imaging (MRI). Furthermore, the evaluation of postoperative complications included cerebrospinal fluid (CSF) leakage and diabetes insipidus (DI).

### Surgical treatment

**Figure 1:**
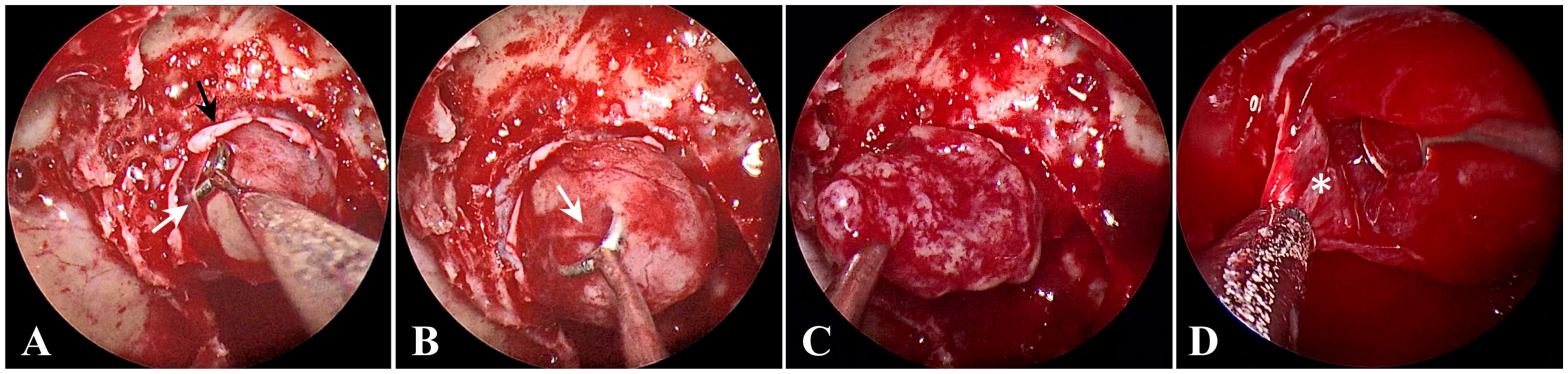
Endoscopic intraoperative views in the areas with pseudocapsule. **(A)** The surgical dissection plane was being established; the opened dura mater (black arrow) and the opened pituitary capsule (white arrow). **(B)** The pseudocapsule (white arrow) was being opened. **(C)** Internal decompression of tumor. **(D)** The remaining tumor shell was removed (white asterisk).

**Figure 2:**
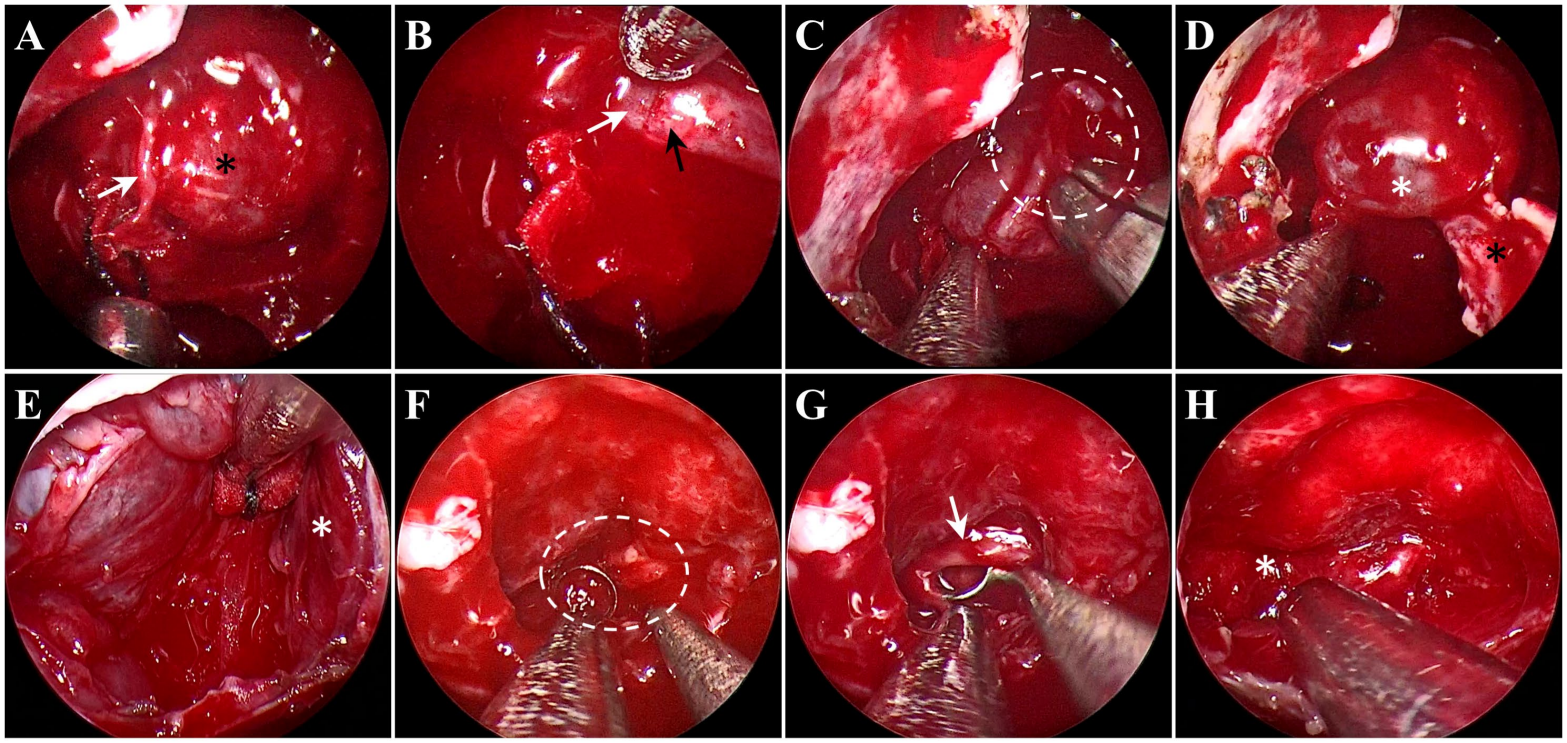
Endoscopic intraoperative views in the areas without pseudocapsule. **(A–E)** Intensive excision bounded by the dural envelope illustrated through case 2. **(A)** There was no pseudocapsule between the tumor tissue (black asterisk) and the pituitary capsule (white arrow). **(B)** The surgical plan established between the pituitary capsule (white arrow) and the diaphragma sellae (black arrow). **(C)** An incision (white circle) was made in the pituitary capsule to control the extent of the pituitary capsule-based extracapsular resection. **(D)** The tumor tissues and pituitary capsule (white asterisk) peeled off from the diaphragma sellae (black asterisk). **(E)** After intensive excision, the pituitary gland (white asterisk) on the left side was preserved. **(F–H)** Intensive excision bounded by the pituitary gland illustrated by case 8. **(F)** No pseudocapsule was detected (white circle). **(G)** The tumor tissues (white arrow) were removed, bounded by the pituitary gland. **(H)** The pituitary gland (white asterisk) was preserved.

For the removal of PitNETs with an incomplete pseudocapsule, the surgical strategy applies the pseudocapsule-based extracapsular resection technique in the areas with pseudocapsule while it adopts the intensive excision bounded by adjacent normal structures in other areas (Supplementary Video S1). Under general anesthesia by endotracheal intubation, the patient was positioned supine with the head tilted slightly to the right. Under the guidance of an endoscope, the middle turbinate was removed to expose the right sphenoid sinus opening, and the bones covering the surfaces of the sellae were resected to reveal the dura mater. It is critical for the removal of PitNETs to identify and utilize the membranous layers surrounding the tumor, including the dural envelope, pituitary capsule, and pseudocapsule. Then, the dura mater was opened widely, while the integrity of the anterior pituitary surface (pituitary capsule) cannot be destroyed. Then, the pituitary capsule would be opened in the area where the pituitary becomes thinner and disappears. In the process of opening the pituitary capsule, the possible pseudocapsule under it should not be destroyed as much as possible. Then, the anterior surface of the tumor was inspected thoroughly to identify whether there was a pseudocapsule. When a pseudocapsule of a tumor is identified intraoperatively, the pseudocapsule-based extracapsular resection technique will be applied. First, it was tried to patiently separate along the outside part of the pseudocapsule to establish a surgical dissection plane (Figure 1A). For the plane can expand smoothly, the pseudocapsule was then opened in the central portion of the exposed tumor (Figure 1B) to remove the central portion of the tumor (Figure 1C) and left with enough thick margin to maintain adequate integrity to the dissection plane. Then, it was continued to patiently expand the surgical dissection plane and remove the remaining tumor shell (Figure 1D). While in the areas without an identifiable pseudocapsule, pseudocapsule-based extracapsular resection cannot be achieved. In this situation, we performed intensive excision with a dissector, blunt ring curette, and aspirator bounded by adjacent normal structures, including the pituitary gland and the dural envelope, to aggressively remove the possible residual tumor cells (Figure 2). In detail, intensive excision bounded by the dural envelope is illustrated through case 2 (Figures 2A–E). When separating the top of the tumor, the surgical plane was interrupted because of the absence of an identifiable pseudocapsule (Figure 2A). Moreover, there was a lack of a recognizable pituitary gland between the tumor tissue and the pituitary capsule. Then, the new surgical plane was established between the pituitary capsule and the diaphragma sellae to peel off the tumor tissues together with the pituitary capsule, which is similar to the extracapsular resection described by Chacko et al. (17) (Figure 2B). Noteworthily, we only circumferentially removed a part of the pituitary capsule in these areas lacking an identifiable pseudocapsule and pituitary gland (Figures 2C,D). The intensive excision was performed to remove the tumor tissues close to the pituitary gland using a blunt ring curette, and the pituitary gland was preserved in the end (Figure 2E). Intensive excision bounded by the pituitary gland is illustrated by case 8 (Figures 2F–H). There was no identifiable pseudocapsule at the top of the tumor (Figure 2F), but the pituitary gland was detected beneath the tumor by surgical exploration and preoperative imaging. Then, the tumor tissues were intensively scraped away bounded by the pituitary gland with a blunt ring curette (Figure 2G). We do not peel off the pituitary gland and avoid too many invasive manipulations of the pituitary gland. The majority of the pituitary gland was preserved in the end (Figure 2H). Finally, the skull base was repaired with the common strategies of using a vascular nasal septal mucosal flap, gelatin sponge, or an artificial dura mater.

### Pathological examinations

Surgically resected tumor tissues were evaluated by routine pathological and immunohistochemical examination. Immunohistochemical localization of transcription factors was carried out, including pituitary transcription factor-1 (PIT-1), steroidogenic factor-1 (SF-1), and pituitary-restricted transcription factor (TPIT). A set of hormone immunostains was used to localize growth hormone (GH), adrenocorticotropin (ACTH), prolactin (PRL), thyrotropin (TSH), follicle-stimulating hormone (FSH), and luteinizing hormone (LH). In addition, a histological examination of these suspicious tissues was also performed using hematoxylin and eosin (H&E) and reticulin staining, which not only evaluated whether they contained residual tumor cells but also detected whether they existed histological evidence of pseudocapsule.

## Results

### Patient and tumor characteristics

Table 1 shows the preoperative conditions of these cases, including age, sex, hormonal type, maximum tumor diameter, Knosp grade, and clinical symptoms. The patient’s ages ranged from 33 to 64 years, including five men and five women. There are three GH-secreting PitNETs and seven non-functioning PitNETs. According to preoperative MRI, all tumors belong to non-invasive PitNETs. They complained mainly of visual impairment and headaches. In total, two patients preoperatively showed partial anterior pituitary dysfunction, including one patient with GH deficiency and one patient with hypothyroidism, and one patient also showed some acromegalic features, including the lengthening of the face and enlargement of the nose, lips, and interphalangeal joints.

**Table 1:** Preoperative characteristics of 10 patients.

No.	Age /Sex	Hormonal type	Maximum tumor diameter (mm)	Knosp grade	Clinical symptoms
1	50/M	NF	21	1	Visual impairment, headache
2	52/F	NF	23	2	Visual impairment, headache
3	37/F	GH	19	0	Headache
4	60/F	NF	20	0	Visual impairment, headache, GH deficiency
5	35/M	NF	17	1	Visual impairment, headache
6	44/M	NF	23	2	Visual impairment, headache
7	33/M	NF	16	0	Visual impairment, Headache
8	56/F	GH	16	0	Headache, acromegalic features
9	61/F	GH	14	0	Headache
10	64/M	NF	21	0	Visual impairment, headache, hypothyroidism

M, male; F, female; NF, non-functioning; GH, growth hormone.

### Surgical outcomes

The postoperative characteristics of all patients are shown in Table 2. In all cases, no residual tumor was detected on postoperative MRI, and gross total tumor resections were achieved. Only two of 10 patients experienced intraoperative CSF leakage, and there was no postoperative CSF leakage in all patients. In total, two patients suffered transient DI after EES, but none developed permanent diabetes insipidus at subsequent follow-ups. Postoperative biochemical remission was achieved in all three somatotroph tumors during the follow-up period. There was no deterioration of pituitary functions postoperatively in all 10 cases. The overall mean follow-up period was 29 months (ranging from 24 to 37 months). At the last follow-up, the patients with preoperative hypopituitarism had improved postoperatively. There was no recurrence during the follow-up period.

**Table 2:** Postoperative characteristics of 10 patients.

No.	EOR	Intraop. CSF leakage	Postop. CSF leakage	Transient DI	Biochemical remission	Pituitary function	Follow-up (months)	Recurrence
1	GTR	Yes	No	No	/	/	24	No
2	GTR	No	No	Yes	/	/	37	No
3	GTR	No	No	No	yes	/	36	No
4	GTR	No	No	No	/	Improved	24	No
5	GTR	No	No	No	/	/	25	No
6	GTR	Yes	No	No	/	/	36	No
7	GTR	No	No	No	/	/	24	No
8	GTR	No	No	No	Yes	/	36	No
9	GTR	No	No	No	Yes	/	24	No
10	GTR	No	No	Yes	/	Improved	24	No

EOR, extent of resection; GTR, gross total resection; Intraop., intraoperative; postop., postoperative; CSF, cerebral spinal fluid; DI, diabetes insipidus.

### Pathological features

**Figure 3:**
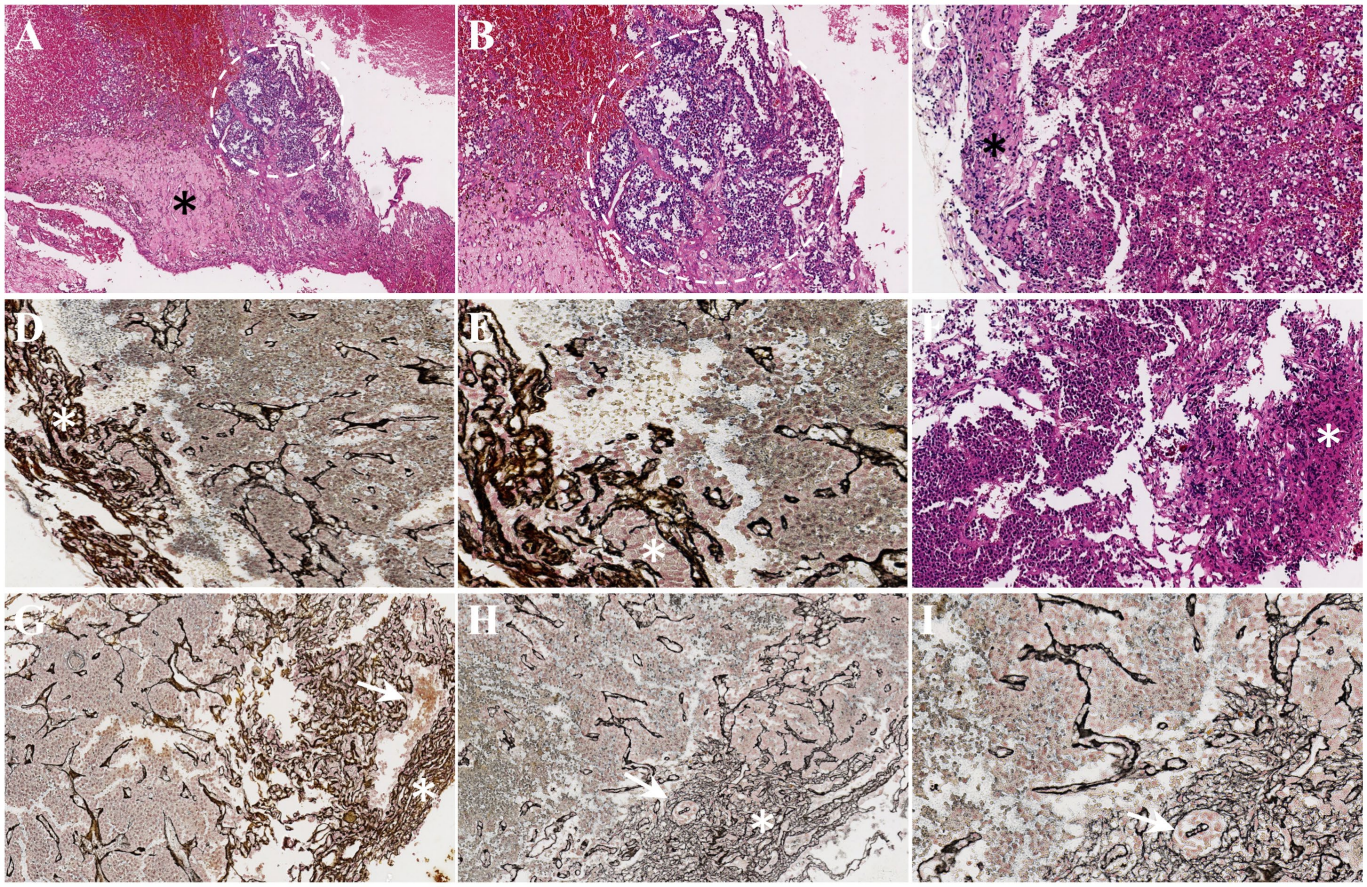
Histopathology of pseudocapsules and the suspicious tissues. **(A)** A thick pseudocapsule (black asterisk) and tumor cell mass (white circle) were detected by H&E staining. **(B)** With higher magnification, the boundary between the tumor cell mass and the pseudocapsule is unclear. **(C)** H&E staining indicated a thin connective tissue as a pseudocapsule (black asterisk). **(D)** Reticulin staining also showed thin pseudocapsule (white asterisk). **(E)** A higher magnification exhibited the clusters of tumor cells (white asterisk) in the pseudocapsule. **(F–I)** The fragmentary pseudocapsule (white asterisk) and tumor cells (white arrow) in the suspicious tissues were identified (**F**, H&E staining; **G–I**, reticulin staining).

The preoperative clinical diagnosis of all patients was confirmed by pathological examination including three somatotroph tumors and seven gonadotroph tumors (Table 3). The pseudocapsule and the suspicious tissues were also examined by H&E and reticulin staining. The histopathology revealed that the pseudocapsules were composed of fibroblasts, collagen fibers, and tumor cells on a background of myxoid materials (Figures 3A–E). By using reticulin staining, clusters of tumor cells were clearly identified in the pseudocapsule (Figure 3E). The pathology of the suspicious tissues showed that tumor cells were detected in all 10 specimens, of which seven samples were composed of fragmentary collagen fibers and tumor cells, two cases consist of the pituitary tissue and tumor cells, and one case was formed from fragmentary collagen fibers, tumor cells, and the pituitary tissue (Table 3). The fragmental pseudocapsule and small tumor remnants were found in the majority of suspicious tissues by histological staining (Figures 3F–I).

**Table 3:** Pathological types of tumors and pathological components of suspicious tissues.

No	Immunohistochemistry	Pathological types	Pathological components		
			Tumor cells	Pituitary tissue	Collagen fibers
1	SF1(+), PIT-1(−), TPIT(−), FSH(+), LH(−), GH(−), ACTH(−), PRL(−), TSH(−)	Gonadotroph tumor	+	+	−
2	SF1(+), PIT-1(−), TPIT(−), FSH(+), LH(−), GH(−), ACTH(−), PRL(−), TSH(−)	Gonadotroph tumor	+	−	+
3	PIT-1(+), TPIT(−), SF1(−), GH(+), ACTH(−), PRL(−), TSH(−), FSH(−), LH(−)	Somatotroph tumor	+	+	−
4	SF1(+), PIT-1(−), TPIT(−), FSH(−), LH(−), GH(−), ACTH(−), PRL(−), TSH(−)	Gonadotroph tumor	+	−	+
5	SF1(+), PIT-1(−), TPIT(−), FSH(+), LH(+), GH(−), ACTH(−), PRL(−), TSH(−)	Gonadotroph tumor	+	−	+
6	SF1(+), PIT-1(−), TPIT(−), FSH(+), LH(−), GH(−), ACTH(−), PRL(−), TSH(−)	Gonadotroph tumor	+	−	+
7	SF1(+), PIT-1(−), TPIT(−), FSH(−), LH(−), GH(−), ACTH(−), PRL(−), TSH(−)	Gonadotroph tumor	+	−	+
8	PIT-1(+), TPIT(−), SF1(−), GH(+), ACTH(−), PRL(−), TSH(−), FSH(−), LH(−)	Somatotroph tumor	+	−	+
9	PIT-1(+), TPIT(−), SF1(−), GH(+), ACTH(−), PRL(−), TSH(−), FSH(−), LH(−)	Somatotroph tumor	+	+	+
10	SF1(+), PIT-1(−), TPIT(−), FSH(−), LH(−), GH(−), ACTH(−), PRL(−), TSH(−)	Gonadotroph tumor	+	−	+

SF-1, steroidogenic factor-1; PIT-1, pituitary transcription factor-1; TPIT, pituitary-restricted transcription factor; GH, growth hormone; ACTH, adrenocorticotropin; PRL, prolactin; TSH, thyrotropin; FSH, follicle-stimulating hormone; LH, luteinizing hormone; +, exist; −, not exist.

## Discussion

The discovery and development of the pseudocapsule represent an important advance in EES for PitNETs. In 1936, the histological capsule surrounding the PitNETs was first observed by Costello at autopsy, and the term “pseudocapsule” was first proposed (18). In 2005, Kawamata et al. described the border between pituitary adenoma and the surrounding normal pituitary tissue as a “microsurgical pseudocapsule” and suggested that its intensive resection is essential (19). Later, in 2006, the term “surgical capsule of the adenoma” was used to describe the histological pseudocapsule between the tumor and the normal pituitary gland by Oldfield and Vortmeyer (20). In addition, laws also used the term “pituitary tumor capsule” to describe the pseudocapsule (21). Although the terms used to describe the boundary between the adenoma and the normal gland are not standardized, the knowledge of the pseudocapsule first described by Costello has been widely recognized. As for the histopathology of the pseudocapsule, Oldfield and Vortmeyer (20) demonstrated that the pseudocapsule was formed by several compressed layers of pituitary acini and their reticulin envelope. Further studies revealed that the pseudocapsule consisted of fibroblasts, collagen fibers, and small cell clusters (22–24). According to reports, the pseudocapsule may also appear yellowish in color or transparent during surgery and may have a hard texture (25). Therefore, the pseudocapsule is developed by the gradual pressure of the tumor on the adjacent normal pituitary gland. According to previous studies, the pseudocapsule was only found in approximately 50% of PitNETs, and many of them are incomplete (26, 27). As for the relationship between the pseudocapsule and tumor size, Costello (18) suggested that the pseudocapsules in smaller PitNETs tended to be complete, while most pseudocapsules in larger tumors might be discontinuous or disrupted. Similarly, Oldfield and Vortmeyer (20) found that the pseudocapsules are easier to be detected in the PitNETs of 2–3 mm in size. Therefore, the formation and development of pseudocapsules are related to tumor size but not positively related. When the PitNET is small, the tumor just began to compress the adjacent normal pituitary tissue without pseudocapsule. As the tumor enlarged in size, the surrounding normal pituitary gland was gradually compressed, and a relatively obvious membranous tissue was produced around the tumor, forming the pseudocapsule. When the tumor grows further, the pseudocapsule will be invaded and destroyed becoming incomplete. In addition, the presence of a pseudocapsule may be related to the endocrine functions of PitNETs. Lee et al. (22) found that the pseudocapsule existed in 70.9% of PRL-secreting PitNETs, 55.0% of GH-secreting ones, 40.0% of ACTH-secreting ones, and in 50.7% of non-functioning tumors. Consequently, the incidence of pseudocapsules depends on whether the PitNETs are functional or non-functional, as well as the endocrinological types of the tumors. In addition, pseudocapsule has also been reported to be associated with the biological behavior and tumor apoplexy of PitNETs (6, 28). In consequence, the pseudocapsule is formed by the pressure of the PitNET on the surrounding pituitary gland and can serve as the boundary between the tumor and the normal pituitary tissue. Therefore, the pseudocapsule can be employed as a surgical dissection plane for removing the tumor while protecting the normal tissue. In 2006, Oldfield and Vortmeyer (20) first tried to dissect the pseudocapsule around the tumor to remove the PitNETs and described the pseudocapsule-based extracapsular resection technique in detail with surgical illustrations. This approach has been widely recognized and used by neurosurgeons for the PitNETs with pseudocapsule, which can completely remove the tumor and obtain excellent surgical outcomes (29–31). However, for PitNETs with incomplete pseudocapsule, there is no consensus on whether and how to remove the suspicious tissue in the regions without pseudocapsule. Before the pseudocapsule-based extracapsular resection technique was developed, there were two other types of extracapsular resection approaches to achieve complete tumor resection: one, initially developed by Wrightson (32), was the removal of the tumor and its covering (including the pituitary capsule, the pseudocapsule, and the dura mater) and the other approach was described by Molitch (16), in which both the tumor and the pituitary capsule are removed while keeping the dura mater, and dissection is performed in a plane between the dura and the pituitary capsule. Nagata et al. (11) performed a peel-off resection of the pituitary gland after selective adenomectomy to remove a small tumor cell remnant in the adjacent pituitary and maximize the effectiveness of EES with a minimal impact on postoperative pituitary function. In this study, we performed an intensive excision bounded by adjacent normal structures to remove the suspicious tissue in the areas without pseudocapsule, where residual tumor cells may be present. The greatest concern in performing the intensive excision is its impact on pituitary functions. It is undeniable that the removal of part of the pituitary capsule will inevitably remove some residual pituitary acini (16). In our study, in fact, the compressed acinar architectures of the pituitary gland were detected in the pathological examination of the suspicious tissues removed by the intensive excision, but the majority of the pituitary gland was preserved in this study. For the intensive excision adjacent to the pituitary gland, we only remove the identifiable tumor tissues with a blunt ring curette. Although we advocate the intensive excision for suspicious tissues in order to remove potential tumor cells, we do not peel off the pituitary gland and also avoid too many invasive manipulations of the identifiable pituitary gland for the protection of pituitary function. Indeed, there was no permanent deterioration of pituitary functions postoperatively in this series, and only two cases suffered postoperative transient DI. Furthermore, preoperative hypopituitarism was improved postoperatively in all patients. In addition, because removing the suspicious tissue in without pseudocapsule areas is more aggressive, the risk of CSF leakage may be greater. According to previous studies, the EES is associated with an intraoperative cerebrospinal fluid (CSF) leakage rate of 20–60% (33, 34) and postoperative leakage rates of 3–15.9% (35, 36). In our surgical series, two cases experienced intraoperative cerebrospinal fluid leakage and no postoperative cerebrospinal fluid leakage in all patients. The previous study has demonstrated that clusters of tumor cells can spread into pseudocapsule at the cellular level (21–23). Noteworthily, in our study, the histopathology of the pseudocapsule revealed that the tumor was partially infiltrated in the pseudocapsule. Therefore, all identifiable pseudocapsules were removed by the pseudocapsule-based extracapsular resection technique. In the areas without pseudocapsule, the tumor cells even invade adjacent tissues, which may lead to tumor recurrence (10, 11). Hence, in these situations, we applied intensive excision bounded by the adjacent normal structures to remove the suspicious tissues in order to remove all potential tumor cells, minimize the risk of PitNETs recurrence, and improve postoperative efficacy. In this study, the fragmental pseudocapsule and small tumor remnants were detected in the majority of these suspicious tissues by histological staining. Therefore, the intensive excision bounded by adjacent normal structures to remove the suspicious tissue in without pseudocapsule areas is an effective and safe surgical strategy for the treatment of PitNETs with incomplete pseudocapsule. However, the limitations of this study are the relatively small sample size. This surgical strategy will continue to be explored by our team in future studies in order to obtain more evidence of its effectiveness.

### Illustrative cases

#### Case 2

**Figure 4:**
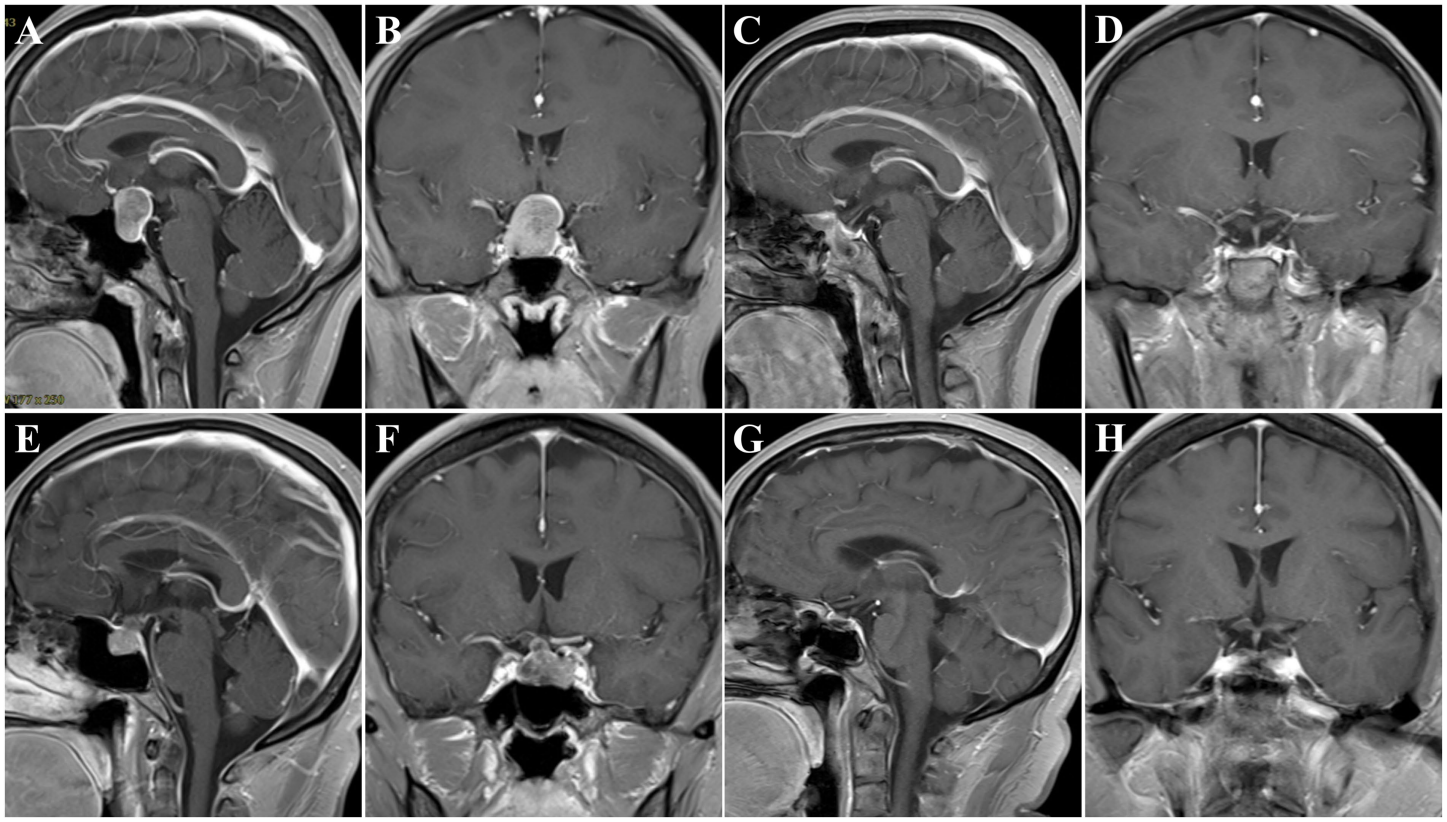
Preoperative and postoperative T1 MR images. **(A–D)** Patient No. 2, preoperative sagittal **(A)** and coronal **(B)** images showing a PitNET, Knosp grade 2. Three-month postoperative sagittal **(C)** and coronal **(D)** images showing GTR. **(E–H)** Patient No. 8, preoperative sagittal **(E)** and coronal **(F)** images showing a PitNET, Knosp grade 0. Three-month postoperative sagittal **(C)** and coronal **(D)** images showing GTR.

A 52-year-old female patient was admitted to our hospital and presented with a headache and visual impairment. On general physical examination, there were no other remarkable symptoms. The endocrinological assessments also disclosed a normal pituitary function. MRI revealed a sellar mass of 2.1 × 1.5 × 2.3 cm which had an elliptical boundary and pushed the optic chiasma to the upward side (Figures 4A,B). In consequence, a clinical diagnosis of non-functioning PitNET was made, belonging to grade 2 on Knosp grade. The tumor was then resected by EES. When a pseudocapsule is identified intraoperatively, the pseudocapsule-based extracapsular resection technique will be applied. While in the areas without pseudocapsule, we applied intensive excision bounded by adjacent normal structures to remove all potential tumor cells. Pathologically, a gonadotroph tumor was confirmed, and the fragmental pseudocapsule and small tumor remnants were found in the suspicious tissues. Postoperative MRI, 3 months later, revealed that gross total resection of the lesion had been achieved (Figures 4C,D). There were no intraoperative complications, but she experienced transient DI after ETSS. Fortunately, she did not turn into permanent DI at subsequent follow-up. Moreover, she had good headache relief, and her vision improved during the follow-up.

#### Case 8

A 56-year-old female patient was admitted to our hospital and presented with intermittent headaches. A general physical examination revealed lengthening of the face and enlargement of the nose, lips, and interphalangeal joints. The endocrinological assessments disclosed a high-level GH of 24.77 ng/mL and IGF-1 of 436.91 ng/mL. Other adenohypophysial hormones were within the normal range. MRI revealed a sellar mass of 1.6 × 1.2 × 1.1 cm (Figures 4E,F). In consequence, a clinical diagnosis of GH-secreting PitNETs was made, belonging to grade 0 on Knosp grade. Then, the tumor was resected by the EES. When a pseudocapsule is identified intraoperatively, the pseudocapsule-based extracapsular resection technique will be applied. While in the areas without pseudocapsule, we applied intensive excision bounded by adjacent normal structures to remove all potential tumor cells. Pathologically, a somatotroph tumor was confirmed, and the fragmental pseudocapsule and small tumor remnants were found in the suspicious tissues. The patient’s serum GH level returned to normal 3 days after the operation. Postoperative MRI, 3 months later, confirmed the gross total resection of the tumor had been achieved (Figures 4G,H). There were no intraoperative or postoperative complications. Moreover, she had good headache relief, and her vision improved during the follow-up.

## Conclusion

We preliminarily explored the effectiveness and safety of the surgical strategy for the removal of the PitNETs without incomplete pseudocapsule. In overview, all identifiable pseudocapsules were removed by the pseudocapsule-based extracapsular resection technique, while the intensive excision bounded by the adjacent normal structures was applied to remove the suspicious tissues in the areas without pseudocapsule.

## Data availability statement

The original contributions presented in the study are included in the article/Supplementary material, further inquiries can be directed to the corresponding author.

## Ethics statement

The studies involving human participants were reviewed and approved by the Ethics Committee of Chongqing General Hospital. The patients/participants provided their written informed consent to participate in this study. Written informed consent was obtained from the individual(s) for the publication of any potentially identifiable images or data included in this article.

## Author contributions

GZ designed the study and drafted the manuscript. PW, JW, DZ, HY, JL, CT, HJ, and XT collected and analyzed the clinical data. NW critically revised the manuscript and contributed to the most important intellectual content. All authors read and approved the final manuscript.

## Conflict of interest

The authors declare that the research was conducted in the absence of any commercial or financial relationships that could be construed as a potential conflict of interest.

## Publisher’s note

All claims expressed in this article are solely those of the authors and do not necessarily represent those of their affiliated organizations, or those of the publisher, the editors and the reviewers. Any product that may be evaluated in this article, or claim that may be made by its manufacturer, is not guaranteed or endorsed by the publisher.

## References

[1] Castle-Kirszbaum M Wang YY King J Kam J Goldschlager T. Quality of life and surgical outcomes in incidental pituitary adenomas undergoing endoscopic endonasal resection. J Neurosurg. (2023) 138:567–73. doi: 10.3171/2022.5.JNS2286, PMID: 35901767

[2] Guo S Wang Z Kang X Xin W Li X. A meta-analysis of endoscopic vs. microscopic Transsphenoidal surgery for non-functioning and functioning pituitary adenomas: Comparisons of efficacy and safety. Front Neurol. (2021) 12:614382. doi: 10.3389/fneur.2021.614382, PMID: 33833725 PMC8021708

[3] Goshtasbi K Lehrich BM Abouzari M Abiri A Birkenbeuel J Lan MY. Endoscopic versus nonendoscopic surgery for resection of pituitary adenomas: A national database study. J Neurosurg. (2020) 134:816–24. doi: 10.3171/2020.1.JNS193062, PMID: 32168478 PMC8080843

[4] Songtao Q Yuntao L Jun P Chuanping H Xiaofeng S. Membranous layers of the pituitary gland: Histological anatomic study and related clinical issues. Neurosurgery. (2009) 64:ons1-9. discussion ons9-10. doi: 10.1227/01.NEU.0000327688.76833.F7, PMID: 19240557

[5] Qu X Xu G Qu Y Song T. The pseudocapsule surrounding a pituitary adenoma and its clinical significance. J Neurooncol. (2011) 101:171–8. doi: 10.1007/s11060-010-0247-0, PMID: 20526794

[6] Wang XB Han TY Ma JG He C Xue L Zhang X. Pseudocapsule and pseudocapsule-based extracapsular resection in pituitary neuroendocrine tumors. Front Endocrinol. (2022) 13:1056327. doi: 10.3389/fendo.2022.1056327, PMID: 36465639 PMC9716262

[7] Chen J Guo X Miao Z Zhang Z Liu S Wan X. Extra-pseudocapsular transsphenoidal surgery for microprolactinoma in women. J Clin Med. (2022) 11:3920. doi: 10.3390/jcm11133920, PMID: 35807204 PMC9267792

[8] Buchfelder M Schlaffer SM Zhao Y. The optimal surgical techniques for pituitary tumors. Best Pract Res Clin Endocrinol Metab. (2019) 33:101299. doi: 10.1016/j.beem.2019.101299 31431397

[9] Qu X Yang J Sun JD Mou CZ Wang GD Han T. Transsphenoidal pseudocapsule-based extracapsular resection for pituitary adenomas. Acta Neurochir. (2011) 153:799–806. doi: 10.1007/s00701-011-0961-1, PMID: 21336808

[10] Zhang X Wang YG Tan J Zhao G Ma M Chen J. Comparison of outcomes between intracapsular resection and pseudocapsule-based extracapsular resection for pituitary adenoma: A systematic review and meta-analysis. BMC Neurol. (2022) 22:52. doi: 10.1186/s12883-022-02574-9, PMID: 35151259 PMC8840683

[11] Nagata Y Takeuchi K Yamamoto T Ishikawa T Kawabata T Shimoyama Y. Removal of the Medial Wall of the cavernous sinus for functional pituitary adenomas: A technical report and pathologic significance. World Neurosurg. (2019) 126:53–8. doi: 10.1016/j.wneu.2019.02.134, PMID: 30849552

[12] Nagata Y Takeuchi K Yamamoto T Ishikawa T Kawabata T Shimoyama Y. Peel-off resection of the pituitary gland for functional pituitary adenomas: Pathological significance and impact on pituitary function. Pituitary. (2019) 22:507–13. doi: 10.1007/s11102-019-00980-w, PMID: 31377966

[13] van Furth WR de Vries F Lobatto DJ Kleijwegt MC Schutte PJ Pereira AM. Endoscopic surgery for pituitary tumors. Endocrinol Metab Clin N Am. (2020) 49:487–503. doi: 10.1016/j.ecl.2020.05.011 32741484

[14] Micko AS Wöhrer A Wolfsberger S Knosp E. Invasion of the cavernous sinus space in pituitary adenomas: Endoscopic verification and its correlation with an MRI-based classification. J Neurosurg. (2015) 122:803–11. doi: 10.3171/2014.12.JNS141083, PMID: 25658782

[15] Melmed S Bronstein MD Chanson P Klibanski A Casanueva FF Wass JAH. A consensus statement on acromegaly therapeutic outcomes. Nat Rev Endocrinol. (2018) 14:552–61. doi: 10.1038/s41574-018-0058-5, PMID: 30050156 PMC7136157

[16] Molitch ME. Diagnosis and treatment of pituitary adenomas: A review. JAMA. (2017) 317:516–24. doi: 10.1001/jama.2016.19699 28170483

[17] Chacko AG Chacko G Seshadri MS Chandy MJ. The ‘capsule’ of pituitary macroadenomas represents normal pituitary gland: A histopathological study. Br J Neurosurg. (2003) 17:213–8. doi: 10.1080/0268869031000153099, PMID: 14565515

[18] Costello RT. Subclinical adenoma of the pituitary gland. Am J Pathol. (1936) 12:205–216.1. PMID: 19970261. PMID: 19970261 PMC1911070

[19] Kawamata T Kubo O Hori T. Surgical removal of growth hormone-secreting pituitary adenomas with intensive microsurgical pseudocapsule resection results in complete remission of acromegaly. Neurosurg Rev. (2005) 28:201–8. doi: 10.1007/s10143-005-0384-7, PMID: 15765245

[20] Oldfield EH Vortmeyer AO. Development of a histological pseudocapsule and its use as a surgical capsule in the excision of pituitary tumors. J Neurosurg. (2006) 104:7–19. doi: 10.3171/jns.2006.104.1.7, PMID: 16509142

[21] Laws ER Jr. Pituitary pseudocapsule. J Neurosurg. (2006) 104:1–2; discussison 2–3. doi: 10.3171/jns.2006.104.1.1 16509140

[22] Lee EJ Ahn JY Noh T Kim SH Kim TS Kim SH. Tumor tissue identification in the pseudocapsule of pituitary adenoma: Should the pseudocapsule be removed for total resection of pituitary adenoma? Neurosurgery. (2009) 64:discussion ons69-70. doi: 10.1227/01.NEU.0000330406.73157.49, PMID: 19240574

[23] Ceylan S Cabuk B Koc K Anik I Vural C. Endoscopic distinction between capsule and pseudocapsule of pituitary adenomas. Acta Neurochir. (2013) 155:1611–9; discussion 1619. doi: 10.1007/s00701-013-1754-5, PMID: 23686633

[24] Zhou Y Wei J Feng F Wang J Jia P Yang S. Pseudocapsule-based resection for pituitary adenomas *via* the endoscopic endonasal approach. Front Oncol. (2022) 11:812468. doi: 10.3389/fonc.2021.812468, PMID: 35111684 PMC8801736

[25] Liu J Ke C Chen X Xu Y Zhang HQ Chen J. Identification and management of suspicious tissue in transsphenoidal pituitary adenoma resection. J Sichuan Univ. (2013) 44:441–3. doi: 10.13464/j.scuxbyxb.2013.03.033 23898531

[26] Kim EH Ku CR Lee EJ Kim SH. Extracapsular en bloc resection in pituitary adenoma surgery. Pituitary. (2015) 18:397–404. doi: 10.1007/s11102-014-0587-4, PMID: 25064083

[27] Jagannathan J Smith R DeVroom HL Vortmeyer AO Stratakis CA Nieman LK. Outcome of using the histological pseudocapsule as a surgical capsule in Cushing disease. J Neurosurg. (2009) 111:531–9. doi: 10.3171/2008.8.JNS08339, PMID: 19267526 PMC2945523

[28] Sugawara T Aoyagi M Tanaka Y Tamaki M Kobayashi D Ohno K. Chronic encapsulated expanding hematoma in nonfunctioning pituitary adenoma. Neurosurg Rev. (2013) 36:395–402. doi: 10.1007/s10143-013-0449-y, PMID: 23345017

[29] Chamoun R Takashima M Yoshor D. Endoscopic extracapsular dissection for resection of pituitary macroadenomas: Technical note. J Neurol Surg A Cent Eur Neurosurg. (2014) 75:48–52. doi: 10.1055/s-0032-1326940, PMID: 23034885

[30] Li QX Wang WH Wang XX. Various strategies of transsphenoidal pseudocapsule-based extracapsular resection in noninvasive functional pituitary adenomas and their effectiveness and safety. Neurol India. (2019) 67:1448–55. doi: 10.4103/0028-3886.273628, PMID: 31857533

[31] Kinoshita Y Taguchi A Tominaga A Arita K Yamasaki F. Pseudocapsular resection in elderly patients with non-functioning pituitary adenoma. Clin Neurol Neurosurg. (2021) 210:106997. doi: 10.1016/j.clineuro.2021.106997, PMID: 34741976

[32] Wrightson P. Conservative removal of small pituitary tumours: Is it justified by the pathological findings? J Neurol Neurosurg Psychiatry. (1978) 41:283–9. doi: 10.1136/jnnp.41.3.283, PMID: 632827 PMC493008

[33] Konuthula N Khan MN Del Signore A Govindaraj S Shrivastava R Iloreta AM. A systematic review of secondary cerebrospinal fluid leaks. Am J Rhinol Allergy. (2017) 31:e48–56. doi: 10.2500/ajra.2017.31.4487 29122076

[34] Shen M Qiao N Shou X Chen Z He W Ma Z. Collagen sponge is as effective as autologous fat for grade 1 intraoperative cerebral spinal fluid leakage repair during transsphenoidal surgery. Clin Neurol Neurosurg. (2022) 214:107131. doi: 10.1016/j.clineuro.2022.107131, PMID: 35134707

[35] Conger A Zhao F Wang X Eisenberg A Griffiths C Esposito F. Evolution of the graded repair of CSF leaks and skull base defects in endonasal endoscopic tumor surgery: Trends in repair failure and meningitis rates in 509 patients. J Neurosurg. (2018) 130:861–75. doi: 10.3171/2017.11.JNS172141, PMID: 29749920

[36] Lee IH Kim DH Park JS Jeun SS Hong YK Kim SW. Cerebrospinal fluid leakage repair of various grades developing during endoscopic transnasal transsphenoidal surgery. PLoS One. (2021) 16:e0248229. doi: 10.1371/journal.pone.0248229, PMID: 33770089 PMC7997021

